# The association between low level exposures to ambient air pollution and term low birth weight: a retrospective cohort study

**DOI:** 10.1186/1476-069X-5-3

**Published:** 2006-02-17

**Authors:** Rose Dugandzic, Linda Dodds, David Stieb, Marc Smith-Doiron

**Affiliations:** 1 Air Health Effects Division, Health Canada, Postal Locator 4602C, Suite 2000, 400 Cooper St., Ottawa Ontario, K1A 0K9, Canada; 2 Perinatal Epidemiology Research Unit, Departments of Obstetrics & Gynecology and Pediatrics, 5850/5980 University Ave, Halifax, Nova Scotia, B3K 6R8, Canada

## Abstract

**Background:**

Studies in areas with relatively high levels of air pollution have found some positive associations between exposures to ambient levels of air pollution and several birth outcomes including low birth weight (LBW). The purpose of this study was to examine the association between LBW among term infants and ambient air pollution, by trimester of exposure, in a region of lower level exposures.

**Methods:**

The relationship between LBW and ambient levels of particulate matter up to 10 um in diameter (PM_10_), sulfur dioxide (SO_2_) and ground-level ozone (O_3_) was evaluated using the Nova Scotia Atlee Perinatal Database and ambient air monitoring data from the Environment Canada National Air Pollution Surveillance Network and the Nova Scotia Department of Environment. The cohort consisted of live singleton births (≥37 weeks of gestation) between January1,1988 and December31,2000. Maternal exposures to air pollution were assigned to women living within 25 km of a monitoring station at the time of birth. Air pollution was evaluated as a continuous and categorical variable (using quartile exposures) for each trimester and relative risks were estimated from logistic regression, adjusted for confounding variables.

**Results:**

There were 74,284 women with a term, singleton birth during the study period and with exposure data. In the analyses unadjusted for year of birth, first trimester exposures in the highest quartile for SO_2 _and PM_10_suggested an increased risk of delivering a LBW infant (relative risk = 1.36, 95% confidence interval = 1.04 to 1.78 for SO_2 _exposure and relative risk = 1.33, 95% confidence interval = 1.02 to 1.74 for PM_10_). After adjustment for birth year, the relative risks were attenuated somewhat and not statistically significant. A dose-response relationship for SO_2 _was noted with increasing levels of exposure. No statistically significant effects were noted for ozone.

**Conclusion:**

Our results suggest that exposure during the first trimester to relatively low levels of some air pollutants may be associated with a reduction in birth weight in term-born infants. These findings have implications for the development of effective risk management strategies to minimize the public health impacts for pregnant women.

## Background

The health impact of exposures to ambient levels of air pollution on susceptible population subgroups such as pregnant women has become an important issue for public health regulators. Several negative reproductive health outcomes have been found to be significantly associated with exposures to air pollutants during pregnancy, including effects on growth, development and duration of pregnancy [1-13]

As low birth weight (LBW) is known to be an important determinant of childhood and possibly adult morbidity [14-16], the public health relevance of this research is evident. Investigations on this important health issue will directly impact on the development of defensible risk management strategies for air pollution. Much of the previous research on this issue has been conducted in regions with relatively high levels of air pollution. In addition, many of the prior studies did not account for important confounders such as maternal smoking. The characterisation of the health impacts of low levels of air pollution from a risk management perspective is especially relevant in terms of the identification of threshold effects.

The province of Nova Scotia is on Canada's eastern coast. According to the 1996 census, 32.9 % of the 1,043,839 residents of Nova Scotia lived in Halifax County, the largest urban area of the province. The primary local sources of air pollution in the province are the industrial sector, generation of electrical power, residential fuel wood combustion and transportation located throughout the province. Nova Scotia, being downwind of some of the larger industrial areas of North America, receives a significant amount of trans-boundary pollution. Environment Canada has estimated that up to 90% of ozone in the province, on high smog days, is attributable to U.S. sources. An estimated 50–70% of the sulfur and nitrogen deposition in eastern Canada is from the eastern U.S. This study was conducted to assess the impact of low level maternal exposures to ozone, sulfur dioxide (SO_2_) and particulate matter of median aerometric diameter less than 10 microns (PM_10_) on fetal growth, according to trimester of exposure.

## Methods

The NovaScotia Atlee Perinatal Database was used to establish a cohort of singleton births between January 1, 1988 and December 31, 2000. The database is a population-based repository of mother/infant information for all live born and stillborn infants that were 500 grams and over at birth. It contains demographic variables, procedures, interventions, maternal and newborn diagnoses, and morbidity and mortality information for pregnancies and births occurring in Nova Scotia hospitals since 1988. Data are collected prenatally, during labor and delivery and postpartum (up to the time of discharge) using standardized data collection forms. After discharge from the hospital, data are abstracted from the medical records by health records personnel and entered into the database. This study was restricted to infants born at term (defined as ≥ 37 weeks of gestation at delivery). Gestational age was based on dates of last menstrual period in the majority of cases and a clinical estimate of gestation when the date of the last menstrual period was unknown. LBW was defined as <2500 grams.

Levels for ozone (O_3_), sulfur dioxide (SO_2_) and particulate matter less than 10 microns in diameter (PM_10_) were obtained from the National Air Pollution Surveillance (NAPS) network. Eighteen monitoring stations provided the ambient air pollution data for the study; only three stations monitored more than one pollutant. Daily data were available for the gaseous pollutants while particulate levels were measured on an every sixth day sampling schedule. There was insufficient ambient monitoring data to examine the impact of other pollutants.

Individual maternal exposure profiles for O_3_, SO_2 _and PM_10_were developed using the resident address reported by the mother at the time of delivery. Geocoding was completed on the basis of postal codes, town or village place name or municipal code. Where possible, the postal code was used to provide latitude and longitude (lat/long) coordinates that could be incorporated into a geographical information system (GIS). For non-rural areas, a postal code represents a relatively specific geographic area. PCCF+/3G (an automated geographic coding system based on the Statistics Canada Postal Code Conversion File) was used to identify lat/long coordinates for each postal code. For rural postal codes, location specificity was obtained by using place name or municipal code for the resident address. The Nova Scotia Gazetteer (NovaScotia Geomatics Center) is a list of 2666 "Status A" place names used in *A Map of the Province of Nova Scotia *and their associated lat/longs. The Gazetteer was used to obtain geocodes when a place name or a specific municipal code provided the most address specificity. If only a rural municipal code was available, a lat/long that corresponded to the average location of all residents was assigned. Though the true distance between rural residences and the central postal location is not known, levels of air pollution in these areas are homogeneous especially given the absence of major industry.

The distance between the mother's address and the closest NAPS station was determined using GIS spatial programming. Only mothers residing within 25 km of a NAPS station were included in the analysis. Trimester exposures were estimated by averaging the ambient pollutant data when a minimum of 75% of the daily values for the period were available. For addresses assigned to more than one monitoring station, the trimester exposure was estimated by distance weighting the pollutant data (1/R^2^) to the station and then averaged over each trimester. This method was chosen to assign exposure levels that give more weight to the monitors that were closer to the woman's residence.

A logistic regression was conducted using SAS Version 8.0 software to assess the relationship between trimester exposures and LBW. Trimester exposures for each pollutant were modeled as both continuous and categorical representations. Relative risks were estimated from odds ratios and 95% confidence intervals calculated. Covariates included in the analysis were maternal age, parity, prior fetal death, prior neonatal death and prior low birth weight infant, smoking during pregnancy, neighborhood family income, infant gender, gestational age, weight change and year of birth. With the exception of neighborhood family income, all of the covariates were derived from the perinatal database. The neighborhood family income is a household size-adjusted measure of household income, based on 1996 census summary data at the enumeration area (the smallest standard census unit consisting of 125 – 375 households) level.

## Results

Between 1988 and 2000 in the province of Nova Scotia, there were 107,331 births ≥ 500 grams among mothers residing within 25 km of a monitoring station. Of these, 89,133 were singleton term births. Trimester exposures were estimated for 74,284 mothers (83%) for whom adequate air monitoring data existed in at least one trimester. Seventy five percent of mothers included in the study resided in an urban region. Thirty-nine percent of mothers were linked to a monitoring station on the basis of postal code; 51% on the basis of place name and 10% on the basis of municipal code. There were no significant differences in covariates between women in the perinatal database who were, and were not, assigned exposure estimates (Table 1).

**Table 1:** Characteristics of eligible cohort members assigned exposures and those who were not assigned exposures.

Covariate		Subjects assigned exposure data N = 74,284	Subjects with no exposure data N = 14,849
Maternal age: Mean (SD)		28.6 (5.2)	28.0 (5.1)
Pre-pregnancy weight: Mean (SD)		64.7 (13.7)	66.5 (14.9)
Weight at delivery: Mean (SD)		79.7 (13.9)	81.0 (14.9)
Infant Gender			
	Male	51.0%	50.6%
	Female	49.0%	49.4%
Nulliparous			
	Yes	45.3%	42.7%
	No	54.7%	57.3%
Smoking at delivery			
	Yes	24.9%	25.8%
	No	75.1%	74.2%
Pregnancy complications			
	Yes	3.9 %	4.1 %
	No	96.1 %	95.9 %
Income Quintile			
	1	21.6%	17.0%
	2	20.0%	20.7%
	3	19.9%	20.2%
	4	21.4%	21.9%
	5	17.1%	20.9%
Urban/Rural residence			
	Urban	74.7%	70.6%
	Rural	25.3%	29.4%

**Figure 1:**
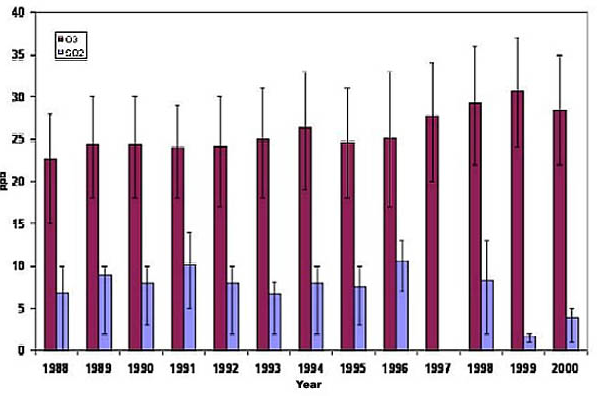
**Annual means, 25^th ^and 75^th ^quartiles of ozone and SO_2 _in Nova Scotia, Canada 1988–2000**. There were no SO_2 _recordings in 1997.

**Figure 2:**
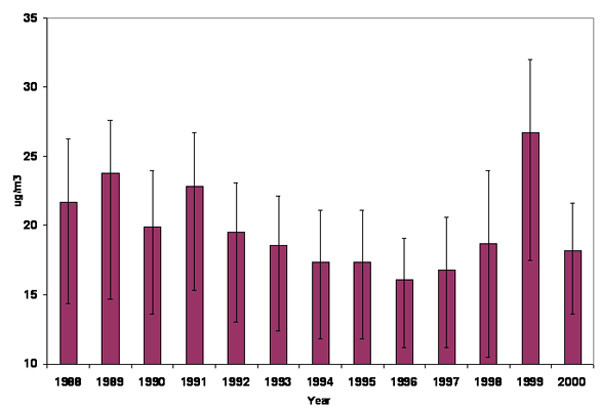
Annual means, 25^th ^and 75 ^th ^quartiles of PM_10 _in Nova Scotia, Canada 1988–2000.

The mean trimester exposures observed for O_3_, SO_2 _and PM_10 _during the study period were 21 ppb, 10 ppb and 17 ug/m^3^, respectively (Table 2). In addition, statistically significant trends were observed for SO_2 _and O_3_, but not PM_10_, in the annual means of each pollutant (Figures 1 and 2).

**Table 2:** Mean Exposures for all trimesters by pollutant, Nova Scotia, 1988 – 2000

Pollutant (24 hour average)	Mean	25%	50%	75%	MAX
Ozone (ppb)	21	17	20	24	43
Sulfur Dioxide (ppb)	10	7	10	14	38
PM_10 _(μg/m^3^)	17	14	16	19	53

During the period of interest in this analysis, the annual rates of term LBW in Nova Scotia ranged from 1.45% to 2.38% with a decreasing trend over time (p < 0.01). Of the study cohort, 1193 (1.6%) term births were classified as LBW. The prevalence of LBW tended to be higher in mothers who were less than 20 years of age at the time of birth, experienced prior fetal or neonatal death or prior low birth weight, smoked during pregnancy and were primipara.

Table 3 illustrates the results by quartile of exposure to O_3_, SO_2_and PM_10 _for each trimester. No significant second or third trimester effects were observed for these 3 pollutants. For SO_2_, statistically significant increased risks of LBW were seen at the highest exposure level during the first trimester (adjusted RR 1.36; 95%CI 1.04–1.78). The inclusion of birth year in the model, however, reduced the magnitude and statistical significance of the effect (RR = 1.26, 95% CI = 0.96–1.66). There was a suggested dose-response effect with increasing levels of SO_2 _during the first trimester. Effect modification was not observed between birth year and quartile of SO_2_.

**Table 3:** Relative risks for term low birth weight according to trimester-specific exposure to air pollutants.

		Unadjusted* RR (95% CI)	Adjusted** RR (95%CI)	Adjusted for Birth Year*** RR (95%CI)
	**1^**st**^Trimester**			
O_3_				
	< 25^th ^percentile (referent)			
	25 – 50^th ^percentile	1.15 (0.89, 1.50)	1.16 (0.88, 1.53)	1.11 (0.83, 1.47)
	51 – 75^th ^percentile	1.11 (0.85, 1.45)	1.04 (0.79, 1.37)	1.02 (0.77, 1.36)
	> 75^th ^percentile	1.06 (1.80, 1.40)	1.01 (0.75, 1.35)	1.00 (0.74, 1.34)
	Continuous****	1.01 (0.90, 1.14)	0.98 (0.86, 1.11)	0.98 (0.86, 1.12)
SO_2_				
	< 25^th ^percentile (referent)			
	25 – 50^th ^percentile	0.93 (0.71, 1.22)	0.96 (0.73, 1.28)	0.98 (0.74, 1.31)
	51 – 75^th ^percentile	1.15 (0.87, 1.52)	1.18 (0.88, 1.58)	1.12 (0.84, 1.50)
	> 75^th ^percentile	1.30 (1.00, 1.67)	1.36 (1.04, 1.78)	1.26 (0.96, 1.66)
	Continuous****	1.17 (1.02, 1.33)	1.20 (1.05, 1.38)	1.15 (1.00, 1.31)
PM_10_				
	< 25^th ^percentile (referent)			
	25 – 50^th ^percentile	1.24 (0.95, 1.62)	1.24 (0.94, 1.64)	1.14 (0.86, 1.52)
	51 – 75^th ^percentile	1.25 (0.96, 1.62)	1.24 (0.95, 1.64)	1.08 (0.82, 1.44)
	> 75^th ^percentile	1.28 (1.00, 1.65)	1.33 (1.02, 1.74)	1.11 (0.84, 1.48)
	Continuous****	1.09 (1.00, 1.18)	1.09 (1.00, 1.19)	1.03 (0.94, 1.14)
	**2^**nd**^Trimester**			
O_3_				
	< 25^th ^percentile (referent)			
	25 – 50^th ^percentile	0.99 (0.76, 1.30)	0.93 (0.70, 1.23)	0.90 (0.67, 1.20)
	51 – 75^th ^percentile	1.13 (0.87, 1.45)	1.07 (0.82, 1.40)	1.07 (0.81, 1.41)
	> 75^th ^percentile	1.01 (0.76, 1.33)	0.98 (0.73, 1.31)	0.98 (0.73, 1.30)
	Continuous****	1.03 (0.92, 1.17)	1.03 (0.91, 1.18)	1.04 (0.91, 1.18)
SO_2_				
	< 25^th ^percentile (referent)			
	25 – 50^th ^percentile	1.14 (0.89, 1.47)	1.12 (0.86, 1.46)	1.14 (0.88, 1.49)
	51 – 75^th ^percentile	1.05 (0.80, 1.37)	1.13 (0.85, 1.50)	1.09 (0.82, 1.45)
	> 75^th ^percentile	0.92 (0.71, 1.20)	1.04 (0.79, 1.37)	0.99 (0.75, 1.32)
	Continuous****	0.93 (0.82, 1.05)	0.99 (0.87, 1.13)	0.97 (0.85, 1.11)
PM_10_				
	< 25^th ^percentile (referent)			
	25 – 50^th ^percentile	0.98 (0.76, 1.28)	1.02 (0.77, 1.34)	0.99 (0.75, 1.31)
	51 – 75^th ^percentile	1.09 (0.84, 1.40)	1.16 (0.89, 1.51)	1.10 (0.84, 1.45)
	> 75^th ^percentile	1.00 (0.77, 1.28)	1.09 (0.83, 1.42)	1.01 (0.76, 1.34)
	Continuous****	1.00 (0.91, 1.09)	1.02 (0.93, 1.12)	1.00 (0.90, 1.10)
	**3^**rd**^Trimester**			
O_3_				
	< 25^th ^percentile (referent)			
	25 – 50^th ^percentile	0.85 (0.64, 1.13)	0.89 (0.66, 1.19)	0.86 (0.64, 1.16)
	51 – 75^th ^percentile	0.94 (0.72, 1.22)	0.95 (0.72, 1.25)	0.94 (0.71, 1.25)
	> 75^th ^percentile	0.89 (0.67, 1.18)	1.01 (0.75, 1.36)	1.03 (0.76, 1.38)
	Continuous****	0.94 (0.83, 1.07)	1.00 (0.87, 1.15)	1.01 (0.88, 1.16)
SO_2_				
	< 25^th ^percentile (referent)			
	25 – 50^th ^percentile	1.05 (0.82, 1.34)	1.04 (0.80, 1.34)	1.03 (0.80, 1.34)
	51 – 75^th ^percentile	0.80 (0.60, 1.07)	0.85 (0.63, 1.15)	0.81 (0.60, 1.10)
	> 75^th ^percentile	0.87 (0.68, 1.13)	0.88 (0.67, 1.15)	0.82 (0.63, 1.08)
	Continuous****	0.91 (0.80, 1.03)	0.93 (0.81, 1.06)	0.89 (0.78, 1.02)
PM_10_				
	< 25^th ^percentile (referent)			
	25 – 50^th ^percentile	0.93 (0.72, 1.20)	0.96 (0.73, 1.26)	0.92 (0.70, 1.21)
	51 – 75^th ^percentile	1.07 (0.83, 1.37)	1.14 (0.88, 1.48)	1.04 (0.80, 1.36)
	> 75^th ^percentile	0.92 (0.71, 1.18)	1.03 (0.79, 1.35)	0.92 (0.69, 1.22)
	Continuous****	0.95 (0.87, 1.05)	0.99 (0.89, 1.09)	0.94 (0.85, 1.05)

* Unadjusted model has only air pollution and no other covariates

** Adjusted for gender of infant, gestational age, maternal age, parity, smoking during pregnancy, weight change, prior neonatal deaths, prior stillbirth, prior low birth weight and neighborhood family income.

*** Adjusted for birth year, gender of infant, gestational age, maternal age, parity, smoking during pregnancy, weight change, prior neonatal deaths, prior stillbirth, prior low birth weight and neighborhood family income.

**** RR for continuous model based on inter quartile range

Mothers in the highest quartile PM_10 _level during the first trimester were found to be at increased risk of delivering a LBW infant in comparison to those exposed to levels < 25^th ^percentile (adjusted RR 1.33; 95%CI 1.02, 1.74), before adjustment for birth year. Adjustment for birth year attenuated this relationship (RR = 1.11) and it was no longer statistically significant (95% CI 0.84–1.48). No evidence of effect modification between birth year and PM_10 _exposure was detected.

Table 3 also shows the results using continuous representations of the three pollutants studied. Adjusted for birth year, an interquartile increase in ppb of SO_2 _exposure during the first trimester was associated with a 15% increase in LBW risk (adjusted RR 1.15; 95% CI 1.00, 1.31) and was of borderline significance. The continuous representations of O_3 _and PM_10 _during the first trimester were not found to be statistically significantly associated with low birth weight.

**Figure 3:**
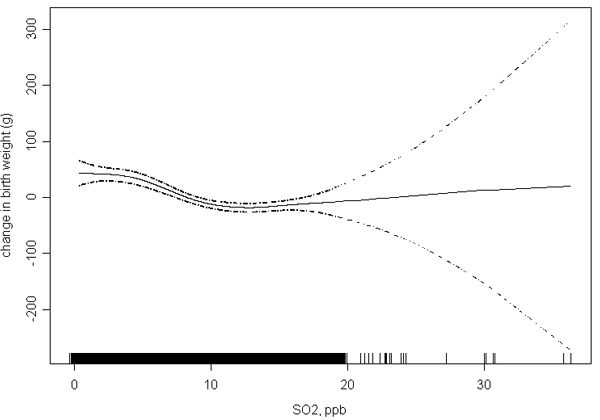
**Change in birth weight versus natural spline function of 1^st ^trimester SO2 exposure**. Dotted lines represent 95% confidence interval and rugplot on x-axis depicts distribution of SO2 exposures.

In order to further assess the shape of the concentration-response relationship, we plotted the relationship between birth weight (as a continuous variable) and a natural spline function of SO_2 _exposure (Figure 3). The results indicate a linear concentration response between increasing level of SO_2_and decreasing birth weight, over most of the range of exposure. The widely divergent error bars beyond 20 ppb reflect the scarcity of observations at higher levels of exposure, as depicted by the rugplot at the bottom of the figure.

The mean birth weight for all exposure categories and for each pollutant was over 3400 grams (data not shown). The largest difference in mean birth weight between the bottom and top quartiles of exposure was seen for first trimester exposure to SO_2 _(3467 grams for bottom quartile versus 3428 grams for those in the top quartile of exposure).

## Discussion

In our analysis of full-term Nova Scotia infants, increased risks of LBW were observed for mothers in the highest exposure quartile for PM_10 _and SO_2 _during the first trimester, unadjusted for birth year. There was a 33% and 36% increase in risk of LBW associated with maternal exposures during the first trimester to the > 75^th ^percentile for PM_10 _and SO_2_, respectively. Adjusting for birth year attenuated the risks, especially for PM_10_. In models based on continuous exposure variables, SO_2 _exposures during the first trimester exhibited a significant association with LBW. Consistent with other studies, we did not observe an increased risk associated with maternal exposure to O_3 _[3,13]. Though adjustment for birth year did attenuate the associations, a dose response relationship for the first trimester was still evident with SO_2. _The plot representing the natural spline function of SO_2 _and birth weight was consistent with a linear concentration response between SO_2 _and birth weight, over most of the range of exposure. This is in keeping with the apparent gradient of effect observed in the logistic model. The lack of statistical significance at the lower exposure quartiles may be a result of low power for observing relatively small increases in risk.

If a causal relationship between air pollutants and birth outcomes is confirmed, a research priority will be to identify the critical time points during pregnancy when exposure to air pollutants might be most harmful [17]. Our finding of an effect with first trimester exposure, but not exposures during the second or third trimesters, is consistent with a number of other investigations. Previous results related to both intrauterine growth retardation (IUGR) and low birth weight suggest that early pregnancy exposures may be the critical time point [1,3,8-10,12,13]. Among the studies that observed a relationship between early pregnancy exposure to air pollutants and IUGR, our exposure levels were not consistently lower or higher than those reported in other studies. For instance, the high exposure group for PM_10 _was ≥ 50 ug/m3 in a study conducted in the Czech Republic [9], whereas the top quartile of PM_10 _in our study was ≥ 19 ug/m3. In Vancouver, British Columbia, the top quartile exposure level for SO_2 _was ≥ 6.3 ppb [13], which was a lower level than our top quartile. Other studies that have reported an effect of air pollutants on birth weight or growth have not observed an effect limited to the first trimester [4,6,7,11]. Biologic mechanisms suggested to support the hypothesis of an effect associated with early pregnancy exposures relate to the etiology of IUGR. Although likely multifactorial, one suggested mechanism for IUGR is abnormal placental development in early pregnancy [18]. Hematologic effects of air pollutants might occur from an initial inflammatory response resulting in increased blood coagulation, and subsequent decreased oxygen supply to the placenta [3,19]. Another hypothesis suggests that the polycyclic aromatic hydrocarbons (PAH) component of PM contributes to impaired growth [20]. Newborns with elevated PAH-DNA adducts (which are used as a proxy to measure individual biologically effective dose to PAH) were found to have significantly reduced birth weight and head circumference suggesting that transplacental exposures to PAHs in ambient air may negatively impact on fetal development [21]. More work is required to fully elucidate the physiologic mechanisms by which air pollution may affect fetal growth and development and to determine if the mechanisms are pollutant specific.

Several limitations of this study are noted. With the existing database, it was not possible to identify multiple births during the observational period for a particular mother. Therefore, clustering or co-linearity could not be accounted for. In addition, there was some concern that classifying exposure based on monitoring sites up to 25 km from the mother's residence could have resulted in significant exposure misclassification. Epidemiology research on the health effects of ambient air pollution is primarily dependent on the proximity of populations to the location of monitoring stations. For the gaseous pollutant, SO_2_, this may be a more relevant issue, as SO_2_concentrations are known to decrease substantially as distance from source increases. Monitor site-pair correlations for SO_2 _across Canada, however, indicate a strong correlation (0.8) for SO_2 _within a distance of 25 km. (Environment Canada). As well, 70% of mothers in our study resided within 15 km of a monitoring station. A recent study in California found that, although neighborhood and county level exposure metrics for PM_2.5 _were highly correlated, the two metrics produced different estimates for the association with birth weight [22]. It is important to note that studies with exposure assignment based on geographical location may result in non-differential classification of the exposure, which can underestimate the magnitude of the association. Thus, inferences across studies that have used varying exposure metrics make comparisons difficult. A recent review of published studies on prenatal and early childhood effects concluded that future investigations should focus on using a more definitive means of characterizing exposures such as personal exposure monitoring to adequately evaluate the impact of each pollutant during different periods of pregnancy [23].

We found that year of birth appeared to be a confounder of the relationship between air pollution and low birth weight, reducing both the magnitude and statistical significance of the association. Our dataset extended over a relatively long period (13 years) during which significant time trends could be detected. Other investigators should evaluate the impact of adjusting for year of birth where extended time periods are under consideration.

As with previous investigations on this issue, there was an inability to adjust for some potentially important confounders such as occupational exposures, exposures to environmental tobacco smoke, maternal drug and alcohol use, or meteorological factors. Similarly, we were unable to account for indoor air exposures such as wood combustion or other housing characteristics. A particular strength of this analysis, however, was the availability of individual-level information pertaining to smoking during pregnancy. However, since the smoking information reflected smoking at the time of delivery, the smoking status for some women may have been underestimated for first (or second) trimester exposures (e.g., a woman who smoked in early pregnancy but quit during pregnancy would be considered a non-smoker).

In this study, it was assumed that maternal residence at the time of delivery was the same residence throughout the pregnancy. Our estimates of trimester specific exposures may be misclassified, particularly for first and second trimester exposures, since the pollutant levels for each trimester were based on residence at delivery. A recent study that evaluated change in residence among pregnant women in Nova Scotia and Eastern Ontario, Canada found that 12% of the women moved during pregnancy and among the women who had changed residence, most moved within the same municipality [24]. Given the small percentage of women who might have moved outside of the municipality between the first trimester and delivery minimizes the likelihood of exposure misclassification due to mobility.

The relationship between exposures to ambient levels of air pollution and birth weight appears to be complex given the somewhat conflicting results that have been observed in terms of pollutant and trimester effects. Whether this represents an inability to accurately characterize exposure for epidemiologic investigations, threshold effects, observed effects being markers of other air pollutants or a result of varying methodologies is not clear. The study region contains significant sources of SO_2 _from the coal based electricity sector and combustion of heavy fuel oils. The disparity in findings may also be partially explained by a combined multi-pollutant effect that is not being captured in the present study designs. In our study, data on other pollutants were not sufficiently available to include in the analysis.

## Conclusion

Consistent with previous observations, this study of exposures to ambient air pollution in Nova Scotia does suggest an association between the highest quartile of exposure of SO_2 _during the first trimester of pregnancy and LBW among term-born infants. Though the data were limited by the inability to assess true exposure burden and the possibility that information on important confounding factors was not available, the study design provides good evidence of a link between maternal exposures to air pollution during the first trimester and fetal outcomes. The ability to adjust for important covariates such as maternal smoking adds strength to the validity of the findings. It is especially pertinent that an association is suggested at relatively low levels of maternal exposure. As the evidence base for maternal exposures to air pollution and adverse reproductive health outcomes becomes more robust, there will be implications for regulators to develop more effective risk management strategies to protect the health of vulnerable populations. The findings from this investigation need to be confirmed with an expanded dataset to more effectively evaluate the multi-pollutant nature of air pollution, including fine particulate matter and the impact of other potential confounders.

## List of abbreviations

CO Carbon monoxide

GIS Geographic information systems

IUGR Intrauterine growth retardation

LBW Low birth weight

NO_2 _Nitrogen dioxide

O_3 _Ground level ozone

PAH Polycyclic aromatic hydrocarbons

PM_10 _Particulate matter less than 10 microns in diameter

ppb Parts per billion

SD Standard deviation

SO_2 _Sulfur dioxide

## Competing interests

The author(s) declare that they have no competing interests.

## Authors' contributions

RD participated in the study design, analysis and drafted the manuscript. LD assisted with study design, writing the manuscript and provided direction with respect to the analysis of the health outcome data. DS provided assistance with the study design, data analysis and interpretation. MS-D carried out GIS linkages between NAPS data and geocoded residences, assigned maternal exposures and conducted statistical analysis. All authors read and approved the final manuscript.
